# Quieting the carrier: Matching delivery to immunology in anti-inflammatory mRNA therapy

**DOI:** 10.1016/j.omtn.2026.103006

**Published:** 2026-07-21

**Authors:** Gabriele Candiani

**Affiliations:** 1 genT_LΛB, Department of Chemistry, Materials and Chemical Engineering “Giulio Natta”, Politecnico di Milano, Milan, Italy

## Main text

Messenger RNA (mRNA) therapeutics have been shaped by two dominant narratives over the past five years. The first, influenced by the success of severe acute respiratory syndrome (SARS)-CoV-2 vaccines and patisiran (Onpattro), has established ionizable lipid nanoparticles (iLNPs) as the *de facto* delivery standard for mRNA. The second asks whether a single delivery archetype can fit every therapeutic intent, particularly when the cargo is meant to dampen immunity rather than provoke it. Philosof et al.^1^ take up precisely this problem. Using a poly(ethylene glycol) (PEG)-free cationic nanoliposome (NLp) to deliver mRNA encoding the ectonucleotidase CD39, they show spatially confined production of active CD39 at a site of acute inflammation, with reduced immune cell infiltration and good short-term tolerability. Their result argues for a straightforward principle: match the carrier to the biology it has to work in, rather than force every cargo into the same vehicle.

Chronic and recurrent inflammation drives a large share of disease worldwide, from atherosclerosis to inflammatory bowel disease (IBD).^2^ While today’s immunomodulatory treatments can be effective, they often work broadly rather than precisely. Drugs like glucocorticoids, methotrexate, colchicine, and biologics such as canakinumab^3^ act throughout the body, which can weaken immune surveillance and create serious trade-offs, a point well illustrated by the increased sepsis-related deaths noted in the Canakinumab Anti-inflammatory Thrombosis Outcomes Study (CANTOS) trial. It is an age-old aim of the research area to develop methods to inhibit inflammation locally without paying a systemic immunological price.

mRNA therapy offers a route to such locality. In view of the fact that mRNA allows transient and dose-tunable protein production inside cells without genomic integration, it is possible in principle to manufacture a therapeutic protein *in situ* for as long as needed.^4^ The bottleneck is delivery. Ionizable LNPs have become the standard against which other carriers are compared, largely because they enabled the clinical breakthroughs of the coronavirus disease 2019 (COVID-19) vaccines.^5^ Yet the very properties that make iLNPs excellent vaccine adjuvants, including engagement of innate sensors, activation of the NLRP3 inflammasome, and induction of IL-6-dependent T follicular helper cell responses,^6^ are precisely those one would wish to avoid when treating an inflammatory disease. Anti-PEG antibodies elicited by repeated mRNA vaccine dosing^7^ add a further translational concern for chronic administration. Non-ionizable carriers, including PEG-free cationic and polymeric systems, have therefore re-emerged as candidates for indications in which carrier-driven immunostimulation is a liability rather than an asset.^5^^,^^8^

The present study contributes directly to this re-examination. CD39 (ENTPD1), the rate-limiting ectoenzyme that hydrolyses extracellular ATP and ADP into AMP, is a natural brake on purinergic inflammation^9^ and a long-standing therapeutic interest of the senior authors’ group, whose recombinant CD39 fusion proteins have shown efficacy in preclinical models of thrombosis, cardiac ischemia, and stroke.^10^ In contrast to previous research, the current investigation examines how the process is altered if one delivers CD39 as a transient mRNA, carried by a vehicle deliberately chosen for its quieter immunological profile.

**Figure 1:**
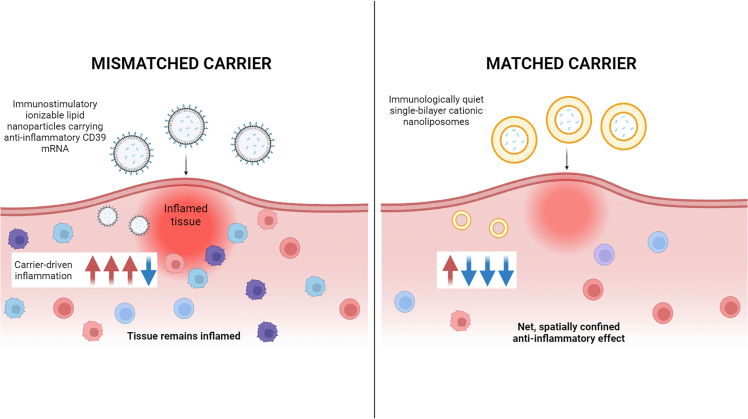
Matching carrier immunology to cargo intent in anti-inflammatory mRNA therapy The same anti-inflammatory cargo (CD39 mRNA) is delivered to inflamed tissue by two carriers with opposite immunological profiles. Left image: an immunostimulatory ionizable lipid nanoparticle (iLNP); innate activation by the carrier counteracts the anti-inflammatory effect of the expressed CD39, so immune infiltration is not reduced. Right image: a PEG-free cationic nanoliposome (NLp), immunologically quiet, allows locally expressed CD39 to act unopposed, producing a net, spatially confined anti-inflammatory effect. Schematic; not to scale. Created in BioRender. https://www.biorender.com/.

The cationic NLps, prepared by microfluidization at a 2:1 1,2-dioleoyl-sn-glycero-3-phosphoethanolamine:3β-[N-(N′,N′-dimethylaminoethane)-carbamoyl]cholesterol (DOPE:DC-Chol) mass ratio, were biophysically well-behaved, unilamellar by cryo-transmission electron microscopy (TEM), with a hydrodynamic diameter near 74 nm, a ζ-potential around +24 mV, and mRNA encapsulation above 90% at sub-neutralization N:P ratios. *In vitro*, NLp/eGFP-mRNA complexes transfected roughly 94% of Chinese hamster ovary (CHO) cells and outperformed a commercial benchmark reagent on mean fluorescence intensity, while NLp/CD39-mRNA produced surface CD39 in both CHO and RAW 264.7 macrophage-like cells and drove ATP hydrolysis in the latter. None of this, on its own, sets the platform apart from many comparable systems. The *in vivo* data are the more telling part. In a subcutaneous (s.c.) Matrigel and lipopolysaccharide (LPS) model, Cy7-labeled NLps remained almost entirely confined to the matrix at 24 h, with only trace signal in liver and kidney and nothing detectable in heart, lung, spleen, fat, or muscle. By day 5, the picture was consistent. Local CD39 mRNA stayed well above every control, CD39 immunostaining rose in plug-derived monocytes, neutrophils, and eosinophils, and *ex vivo* ectonucleotidase activity was measurable. Wall thickness had returned to sham levels, while monocyte and macrophage infiltration sat well below the vehicle and empty-NLp controls, though still above sham. Liver enzymes held steady, and hematology in the treated animals matched controls. The sharpest result is the head-to-head with an Onpattro-like iLNP at the same CD39 mRNA dose: the iLNP produced no measurable reduction in inflammatory cell infiltration. If it holds up, this is strong evidence that the iLNP architecture is not right for every cargo, and that in an inflamed tissue the carrier’s own immunostimulation can cancel an immunosuppressive payload, a kind of pharmacological self-cancellation (Figure 1).

The work is a concrete proof-of-principle for anti-inflammatory mRNA produced locally, within an immune-active tissue compartment, which is a genuinely different proposition from systemic dosing. It also makes a credible, if not yet decisive, case that carrier choice for anti-inflammatory mRNA should follow immunological compatibility with the payload, not transfection efficiency alone. And it adds to the view of CD39 as a tractable enzymatic switch in the purinergic axis, its activity re-established *in situ* from an mRNA template at clinically plausible local doses.

None of this comes without caveats. The cytokine readouts are softer than the histology: the IL-6 reduction missed significance (*p* = 0.06), and TNF-α was unchanged at day 5, in line with the late endpoint but leaving CD39’s molecular signature only partly resolved. A single iLNP comparator is informative but cannot stand for the wider iLNP design space. The Matrigel and LPS model is, by design, an acute, sterile, externally triggered insult, far from the chronic, self-sustaining inflammation of atherosclerosis, IBD, or rheumatoid synovium. There is also no comparison with recombinant CD39 protein, arguably the most informative benchmark against the senior authors’ own earlier work.^10^ And the cationic-lipid architecture, reasonable at the doses used here, still carries concerns about complement engagement, biodistribution on scale-up, and repeat-dose tolerability that need systematic study.

Where might this lead? The natural near-term programmes are image-guided or catheter-directed local administration to vascular or articular inflammation, where confinement of the therapeutic to a defined depot is both pharmacologically desirable and clinically tractable. CD39 mRNA is also a plausible candidate for second-hit prevention after revascularization, or as a complement to induction with monoclonal anti-inflammatories.

More broadly, the conceptual provocation of this paper, that mRNA carriers should be selected to match the immunology of their cargo rather than be imposed on it, should extend well beyond CD39. Non-ionizable cationic, polymeric, and stimuli-responsive systems, long overshadowed by iLNPs, may well find their best translational footholds precisely in immunologically delicate indications. Philosof and colleagues have not closed this question. They have, valuably, reopened it.

## Declaration of interests

G.C. is co-founder and President of Vectorialis S.r.l., a Politecnico di Milano spin-off developing the VibroFect non-viral nucleic acid transfection platform, and is a named inventor on patents covering related technology (EP4153761B1, US12359157B2, WO2021234136A1). His interests lie in non-viral nucleic acid delivery, the same broad field as the technologies discussed here. G.C. has no financial relationship or collaboration with the authors, their institutions, or the specific products evaluated in the commented work. The views expressed are solely those of the author and do not reflect the views, opinions, or position of the employer or any subsidiaries.
